# Gender Differences on Motor Competence in 5-Year-Old Preschool Children Regarding Relative Age

**DOI:** 10.3390/ijerph18063143

**Published:** 2021-03-18

**Authors:** Rubén Navarro-Patón, Joaquín Lago-Ballesteros, Víctor Arufe-Giráldez, Alberto Sanmiguel-Rodríguez, Carlos Lago-Fuentes, Marcos Mecías-Calvo

**Affiliations:** 1 Facultad de Formación del Profesorado, Universidade de Santiago de Compostela, 27001 Lugo, Spain; ruben.navarro.paton@usc.es; 2 Facultad de Ciencias de la Educación, Universidad de A Coruña, 15008 A Coruña, Spain; v.arufe@udc.es; 3 Facultad de Lenguas y Educación, Universidad Camilo José Cela, 28692 Madrid, Spain; asrgz2014@gmail.com; 4 Facultad de Lenguas y Educación, Universidad Nebrija, 28015 Madrid, Spain; 5 Facultad de Ciencias de la Salud, Universidad Europea del Atlántico, 39011 Santander, Spain; carlos.lago@uneatlantico.es (C.L.-F.); marcos.mecias@uneatlantico.es (M.M.-C.); 6 Centro de Investigación y Tecnología Industrial de Cantabria (CITICAN), 39011 Santander, Spain

**Keywords:** relative age effect, childhood, motor competence, manual dexterity, aiming and catching, balance, movement assessment battery for children-2 (MABC-2)

## Abstract

The aim of this study was to evaluate the differences on motor competence between 5-year-old boys and girls and to investigate the existence of Relative Age Effect (RAE) on their motor competence. A total of 232 preschool children were evaluated of whom 134 (57.8%) were boys and 98 (42.2%) were girls. The Movement Assessment Battery for Children-2 (MABC-2) was used to collect data. The data show a main effect on gender factor; there was a main effect in total score of manual dexterity (MD; *p* = 0.010), in total score of balance (Bal; *p* < 0.001), in total test score (TTS; *p* < 0.001), and in total percentile score (TPS, *p* < 0.001). In the semester of birth factor, there were differences in aiming and catching (A&C, *p* < 0.001), in Bal (*p* = 0.029) and in total percentile score (TPS, *p* = 0.010). Girls perform better in MD, Bal, TTS, and TPS than boys. Preschool children born in the first semester obtain, in general, a higher percentage and a higher percentile than their peers born in the second one. RAE is present in A&C, Bal, and TPS, with higher scores obtained by preschool children born in the first semester compared to those born in the second one.

## 1. Introduction

A correct work in Physical Education (PE) leads to an improvement in the motor competence of children [1]. Motor competence is known in the motor behavior literature as the ability of each individual to expertly perform different fine and gross motor skills [2], while showing adequate levels of quality and control of movement [3] and the acquisition and improvement of these skills [4].

Basic motor skills are classified in the motor behavior literature as follows: (1) locomotor skills (e.g., running, sliding, jumping); (2) object manipulation and control skills (e.g., hitting, kicking, throwing, catching); and (3) stability and body control skills (e.g., balance, body rocking) [5].

The first years of the individual’s life (0 to 5 years) are a crucial period for children to consolidate as physically competent since the different components of motor competence are the pillars where the most complex movements are based [6,7]. For this reason, the field of motor competence has become a growing area of research of special importance due to its relationship with the health and development of children [8,9] with better cognitive development, with executive functions and with academic performance [9,10,11,12,13,14]. The development of motor competence during childhood is dependent on biological (i.e., genetic, sexual, and maturational) and environmental factors (i.e., stereotypes or gender roles), as well as the relationships between these [15]. 

With respect to gender, it is known that boys and girls go through the same sequence of motor competence development. However, in childhood, girls tend to show lower levels of global motor competence and object control, and manipulation skills than boys [16,17,18,19,20] and, in contrast, girls show a better performance than boys in fine motor skills [21,22,23] and in balance [19,21,22,24,25]. However, during the preschool period (3 to 6 years), it is likely that there is a wide range of individual differences in the development of motor competence between different ages and genders [22,26] and that these differences do not remain stable throughout the preschool period [22].

These differences may be due to relative age, since it has been shown that the size of the Relative Age Effect (RAE) in school evaluations, specifically, in motor competence, has an inverse correlation with age [27]. Available studies show that children born in the first semester of the academic year have better cognitive performance and motor competence than younger children born in the last semester of the same year [28,29,30], in addition to being more likely to obtain higher grades in PE [31]. These advantages of relatively older students may be motivated by differences in physical size and maturation [4,32,33]. Physical maturity could offer older students an advantage, which is sometimes mistaken for superior ability [34].

However, these types of gender effects are not reported in any study on RAE in research related to motor competence at an early age. Few studies are currently found investigating the influence of relative age on general skills and performance measures [29,35,36,37]. Typically, age-relative examinations in youth populations have focused on sports samples, high-performance samples, or elite athletes. The main objective of this study was to analyze the effect of gender and RAE in a sample of 5-year-old children from Galicia (Spain) in tests of manual dexterity, throwing and catching and balance, as well as in the global score in motor competence, hypothesizing that the motor competence, measured by the MABC-2 battery, would be higher in preschool children who were born in the first semester of the year compared to those who were born in the last semester of the same year and concluding there will be gender differences.

## 2. Materials and Methods

### 2.1. Study Design

For the development of this research, a descriptive design was established [38], in which each of the three motor skills that make up the Movement Assessment Battery for Children-2 (MABC-2) have been established as dependent variables of the study (i.e., Manual Dexterity, Aiming and Catching, and Balance) while comparing them based on the independent variables in this study (gender and semester of birth).

### 2.2. Participants

The selection of the participants was carried out through a non-probabilistic procedure, using a sampling by convenience and geographical proximity to choose 5 educational centers of the public-school network of Galicia (Spain).

The inclusion criteria to participate in the study were: (1) provide informed consent signed by their parents or legal guardians; (2) not suffer from illness or difficulty (physical or mental) that prevents participation in the development of the MABC-2 tests; (3) not having a final score below the 5th percentile after the test, since below this percentile, children can have motor competence problems, thereby altering the results.

A total of 248 schoolchildren (5 years old) were invited; overall, 16 were excluded ex post for presenting significant movement difficulties, since they were below the 5th percentile of the battery. Finally, the sample consisted of 232 schoolchildren.

All were classified by semesters based on their date of birth (semester 1 (S1: born from January to June); semester 2 (S2: born from July to December)) and according to gender (boys and girls).

### 2.3. Tools

Sociodemographic data such as date of birth, age, and gender have been collected through ad hoc questionnaires.

For motor skills, the version adapted to the Spanish context by Graupera and Ruíz [39] of the Movement Assessment Battery for Children-2 (MABC-2) [40] was used. It is a valid and reliable test to identify changes in motor competence in preschool children [39,40,41,42] with very high inter-rater reliability [43]. This battery allows knowing the motor competence in three specific skills through eight standardized tests: (1) manual dexterity: posting coins, threading beads (both scored as the time in seconds taken to complete), and drawing trail (scored by the number of errors the subjects make); (2) aiming and catching: catching a bean bag, and throwing a bean bag onto mat (both scored by the number of successful attempts), and (3) balance: one-leg balance (scored as the time recorded), walking on raised heels and jumping on mats (both scored as the number of correct attempts registered) [40]. In this sense, a high score in the different elements of this battery has a positive meaning (less difficulty in carrying it out), so the higher the score, the greater the motor competence [44].

Through this battery, three motor skills are evaluated: manual dexterity (MD), aiming and catching (A&C) and balance (Bal). To obtain the scores for each of them, a total of eight tests must be developed: inserting coins, threading beads and drawing trace (MD); catching the bag of chickpeas and tossing the bag of chickpeas on the mat (A&C); swinging one leg, walking with raised heels and jump on mats (Bal). For each test, direct scores are obtained that must be transformed into scalars and, subsequently, for the calculation of each general motor ability (i.e., MD, A&C, and Bal), the scalar and percentile scores are calculated. With these scores of each motor skill, the overall score of the battery was calculated for each subject (scalar and percentile).

### 2.4. Procedures

First, the educational centers and teachers were contacted explaining the objectives of the study. Once the approval was given, the information and informed consent sheet was sent to the parents or legal guardians of the study participants for their signature and return. Subsequently, the data necessary to carry out the investigation was recorded; sociodemographic data (i.e., age, date of birth, gender) and data from the MABC-2 battery.

For the development of the MABC-2 battery, the general rules of application of the MABC-2 battery of the manual were followed. Standardized material was used and the evaluators (previous training on battery management) assessed the students individually and carried out the same methodology in all educational centers to avoid researcher bias: description of the task, demonstration by the examiner, practice by the child of the test as indicated in the procedure (where the examiner could correct possible errors) and execution of the test following the instructions of the manual (no instructions were given during the test performing). Before carrying out the test, the children were allowed to practice each test to correct possible errors, since during the test, the examiners did not transmit instructions.

The application of the MABC-2 battery was carried out in classrooms provided by the educational centers that participated in the study, which were isolated, bright, clear, well ventilated, and isolated from noise that could produce disturbances or interferences in the evaluation. In addition, for the tests that required sitting (i.e., manual dexterity), the room had a table and two chairs.

For the treatment of the data obtained through the application of the MABC-2 battery, the scalar scores have been calculated for each of the 8 tests and in this way to be able to calculate the scores (scalar and percentile) of each motor skill and the global battery.

The research was approved by the Ethics Committee of the national platform Educa (code 22019), under the standards established in the Declaration of Helsinki.

### 2.5. Statistical Analysis

The variables of the sociodemographic data were expressed in frequencies for the categorical variables and in measures of central tendency (mean and standard deviation) for the quantitative variables. To determine the differences between all the variables of the MABC-2 battery, the semester of birth (S1 and S2) and gender (boys vs. girls), a MANOVA was performed with the Bonferroni statistic to know the significance of the interaction between the variables while knowing the effect size, partial eta squared (η^2^) was used.

To know the differences in the total percentile of the MABC-2 battery between the semester of birth (S1 and S2) and gender (boys vs. girls), a Student’s *t*-test was used for independent samples.

All statistical analyses were carried out using SPSS software (v.25, IBM Corporation, New York, NY, USA). The level of statistical significance was set at *p* < 0.050.

## 3. Results

A total of 232 healthy preschool children were evaluated, of which 134 (57.8%) were boys and 98 (42.2%) were girls. The distribution of the participants by semester of birth was 120 (51.7%) for S1 and 112 (48.3%) for S2. Table 1 shows the results globally, by gender and by semester of birth in each of the specific skills that have been assessed using the MABC-2 battery.

**Figure 1:**
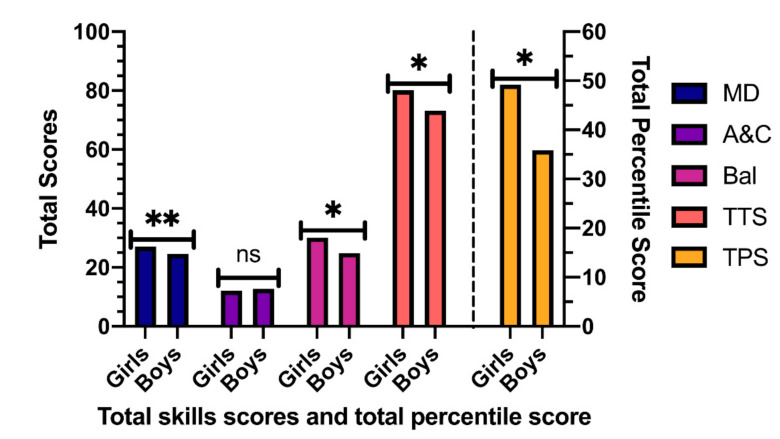
Total skill scores and total percentile score according to gender. MD: Manual dexterity; A&C: Aiming and Catching; Bal: Balance; TTS: Total Test Score; TP: Total Percentile. Note: * *p* < 0.001 different between Girls vs. Boys; ** *p* < 0.05 different between Girls vs. Boys; ns *p* > 0.005 no significative differences.

**Figure 2:**
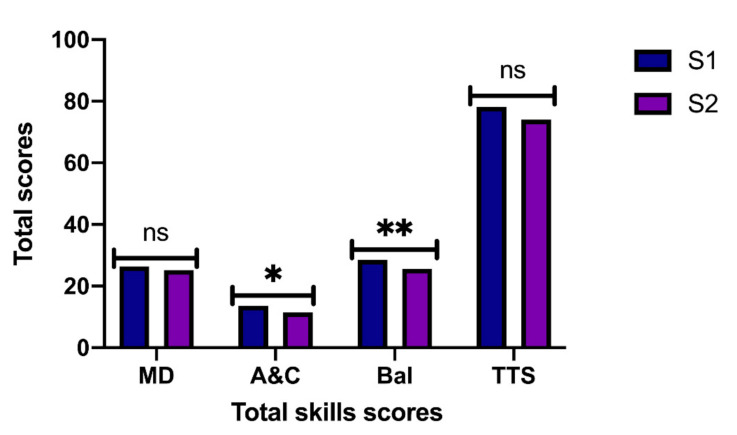
Semester of birth according to total skill scores. MD: Manual dexterity; A&C: Aiming and Catching; Bal: Balance; TTS: Total Test Score. Note: * *p* < 0.001 different between S1 vs. S2; ** *p* < 0.05 different between S1 vs. S2. ns *p* > 0.005 no significative differences.

The results of the MANOVA (Figure 1 and Figure 2) regarding manual dexterity (MD) indicated that there is a significant main effect of the gender factor [*F* (1, 228) = 6.703, *p* = 0.010, η^2^ = 0.03], being higher in girls. A significant effect was also found in the interaction between both factors [*F* (1, 228) = 7.07, *p* = 0.008, η^2^ = 0.03] but not in the factor semester of birth [*F* (1, 228) = 1.983, *p* = 0.160, η^2^ = 0.01].

In terms of aiming and catching (A&C), the findings indicated that there is a significant main effect of the semester of birth factor [*F* (1, 228) = 13.024, *p* < 0.001, η^2^ = 0.05], the scores being higher in those born in the first, but not in the gender factor [*F* (1, 228) = 0.899, *p* = 0.344, η^2^ = 0.01], nor in the interaction of both factors [*F* (1, 228) = 1.803, *p* = 0.181, η^2^ = 0.01].

Regarding balance (Bal), the results of the MANOVA indicated that there is a significant main effect of the gender factor [*F* (1, 228) = 26.712., *p* < 0.001, η^2^ = 0.10] with the highest scores in the girls. There was also found a main effect in the semester of birth factor [*F* (1, 228) = 4.822, *p* = 0.029, η^2^ = 0.02] but not in the interaction between both factors [*F* (1, 228) = 0.674, *p* = 0.412, η^2^ = 0.003].

The results regarding the total test score (TTS) indicated that there is a significant main effect of the gender factor [*F* (1, 228) = 13.338, *p* < 0.001, η^2^ = 0.05] with girls obtaining the highest scores, but not in the semester of birth factor [*F* (1, 228) = 3.654, *p* = 0.057, η^2^ = 0.02], nor in the interaction between semester and gender [*F* (1, 228) = 1.164, *p* = 0.282, η^2^ = 0.01].

Regarding the pairwise comparison in MD (Table 1), statistically significant differences have been found between boys and girls in the first semester (*p* < 0.001), with higher scores in girls, but not in the second semester (*p* = 0.962). Regarding A&C, no statistically significant differences have been found. When Bal is analyzed, statistically significant differences have also been found between boys and girls, in favor of girls in the first semester (*p* = 0.002), as well as in the second semester (*p* < 0.001). Regarding the TTS, in the results of the first semester higher scores appear in girls (*p* = 0.001).

In the analysis by pairs, according to gender and semester of birth, differences were found in girls between the first and second semester in DM (*p* = 0.008) and A&C (*p* = 0.001), being greater in those born in the first semester. In boys, differences were found in Bal (*p* = 0.020) in favor of boys born in the first semester. Regarding the TTS, no significant differences were found.

**Figure 3:**
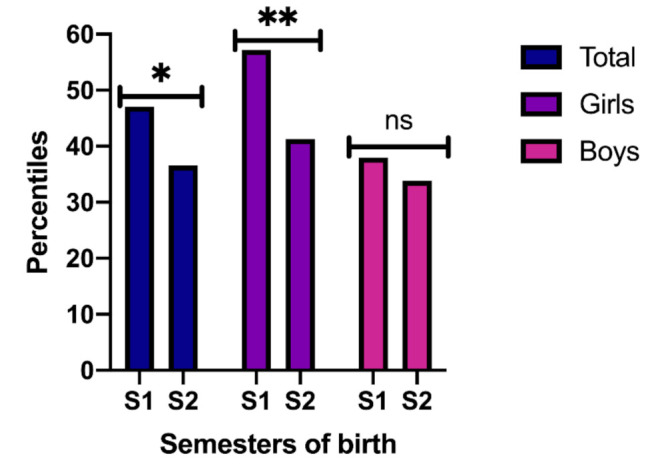
Percentiles according to gender and Semester of birth (S) and global. Note: * *p* < 0.007 different between Semester 1 vs. Semester 2. ** *p* < 0.005 different between Semester 1 vs. Semester 2. ns *p* > 0.005 no significative differences.

Although there are differences between the percentile reached by boys and girls when the semesters are compared (Figure 3), the trend within each gender group indicates that there are statistically significant differences between students born in S1 (M = 47.08, SE = 2.55) vs. S2 (M = 36.56, SE = 2,93; *p* = 0.007). The same is true for girls, S1 (M = 57.16, SE = 3.32) vs. S2 (M = 41.28, SE = 4.62; *p* = 0.005), but it is not true for boys, S1 (M = 37.95, SE = 3.45) vs. S2 (M = 33.84, SE = 3.77; (*p* > 0.050).

## 4. Discussion

The objective of this study was to analyze the effect of gender and RAE in a sample of 5-year-old children from Galicia (Spain) in tests of manual dexterity, throwing and catching, and balance, as well as in the global score in motor competence.

The data obtained in this research indicate that gender statistically significant differences were found between boys and girls. All the scores and percentiles obtained by girls were higher; results that do not support some investigations [16,17,18,19,20,21,22,23,25,45,46], which indicate that boys obtain higher scores globally. These global differences with respect to gender could be due, in part, to the higher scores obtained by girls in the MD [22,47,48] and in the Bal [22,24,37,49], perhaps produced by the development of practices of stereotyped activities, and different sports activities, between boys and girls. Therefore, PE classes should promote coeducational sports activities to try to eliminate these gender differences.

In terms of RAE, those born in S1 (both boys and girls) also obtain higher scores compared to those born in S2, that is, the former are in a higher percentile than the latter [22,36,37,50]. Thus, preschool children born in S1 achieve a higher TPS than their peers born in S2, which can cause those born earlier (S1) to obtain better grades in PE due to achieving better results in standardized physical and motor tests, regardless of the influence of the RAE.

For the specific skills evaluated with the MABC-2 battery, the results of our study on manual dexterity (MD) show that girls obtain higher scores than boys [19,21,22,23,25,37,47,48], in general, and in S1 in particular [36,37], while boys present the same scores as girls in S2. However, our study did not take into account other variables such as the practice of extracurricular activities or the sociocultural environment. There are authors who postulate that this could be related to the type of stereotyped activities or gender role models that girls perform, which are those that make them better in fine motor skills [14], such as writing [20] or practicing hand-eye activities and good coordination [51]. A study found that they present greater perceived manipulation but without finding statistically significant differences in perceived locomotion, perceived manipulation, perceived gross motor performance, and perceived motor competence, according to geographic region and gender [52].

Regarding the semester of birth, no differences were found in this dimension, specifically, when comparing within the same gender [37]. In MD, the same trend is maintained as in the previous tests, that is, the scores of girls born in S1 are higher than the scores of those born in S2. However, this trend changes in boys, with the scores of boys born in S2 being higher compared to those born in S1. These results coincide with those obtained by girls and follow the same line as studies on motor performance [53,54], or selection and detection of sports talent [55,56], where those born in the first months of the year have better motor performance and are the first chosen.

Regarding the A&C, there are no statistically significant differences between genders, with the children’s scores being similar. This is not in agreement with other studies which reveal that boys tend to show higher levels than girls in mobile control and manipulation skills [16,17,18,25,45,46,47,57,58]. However, children (both boys and girls) born in the first semester perform better than their second semester peers, like other studies [21,22,23]. Regarding the semester of birth, and specifically in the A&C, statistically significant differences have also been found, with the scores of those born in the first semester being better, with the RAE being more evident at these ages for the motor competence tests [36,37]. In the comparison of A&C between genders, statistically significant differences are observed in girls when comparing the semesters (in favor of those born in the first semester), while in boys no similar differences are found. When comparing gender and birth semester, it is observed that there are no statistically significant differences in the comparison between boys and girls.

In the Bal, differences have been found with respect to gender, with girls obtaining better scores, regardless of the semester of birth [19,21,22,24,25,37]. This could be due to the fact that girls may have an advantage in the development of postural control [21,22,23] although it is known that this capacity does not finish consolidating until 8–9 years [51]. Regarding the semester of birth, statistically significant differences were found in the comparisons in the Bal being the highest scores obtained by girls in both the first and second semesters [37]. These results could be due to the fact that the development of motor competence during infancy, childhood, and adolescence depend on biological factors [i.e., genetic, sexual and maturational] and environmental [i.e., gender roles, experiences, motor play opportunities (variety of play materials and appropriate physical spaces)] [5,59,60], and their interactions [15].

## 5. Conclusions

It is concluded that there are differences between 5-year-old boys and girls in terms of motor competence. Those children born in S1 achieve better scores in motor competence compared to those of S2 by gender and globally, and girls perform better than boys (i.e., MD, Bal, and TTS). In this sense, the difference of 6 months in the date of birth in preschool children can represent up to 8% of their life [53]. From an educational point of view, PE teachers, must take it into account when scheduling, which should be done in the short term to suit the needs of each child, because of the first years of life (0 to 5 years) are a fundamental period for the development of physically competent children [6,7].

On the other hand, this research should be taken into account by PE teachers, since the evaluation of this subject is usually carried out through standardized tests, which could benefit those born in the first trimesters and harm the youngest within of the same cohort. In this sense, it is known that those students with lower results in motor competence tend to have less participation in organized games or sports during childhood, which leads to a negative spiral of disengagement from an active lifestyle.

In addition, early childhood education centers and schools must play a leading role in providing and promoting equal motor opportunities. Boys and girls spend a large part of their time in schools, in such a way that planned and well-defined programs are implemented from there as a strategy for a harmonious development of motor competence, through pedagogically structured PE classes taught by committed professionals.

## 6. Study Limitations and Future Proposals

Regarding the limitations of this study, our results must be understood within the sociocultural context where the study was developed (Galicia, Spain), and cannot be extrapolated to other regions or countries due to their social and cultural peculiarities. Furthermore, the sample is not sufficiently representative and large; therefore, the results must be taken with caution in order to be generalized. On the other hand, we did not analyze to take into account other factors that could affect RAE at these ages, such as sports attendance or anthropometric parameters [61]. In addition, a product-oriented evaluation tool has been used, that is, the movement is evaluated from a quantitative approach, evaluating the result of the execution and not the process.

Future studies should delve into the effect of relative age in other educational stages within the area of PE and school sports.

## Figures and Tables

**Table 1:** Results of MABC-2 test based on gender and the semester of birthdate.

		Semester 1 (Born from January to June)		Semester 2 (Born from July toDecember)	
Total Scores	Gender	Mean	SEM	Mean	SEM
Manual dexterity (Post coins; Threading beads; Drawing trail 1)	boys	24.04	0.92	25.21 *	0.86
	Girls	28.92 **	0.91	25.14 *	0.87
	Total	26.36	0.68	25.18	0.39
Aiming and catching (Catching bean bag; Throwing bean bag onto mat)	boys	13.49	0.66	11.98	0.58
	Girls	13.75	0.69	10.46 *	0.60
	Total	13.61	0.47	11.42 *	0.43
Balance (One-leg balance; Walking heels raised; Jumping on mats)	boys	26.44	0.77	23.38	0.97
	Girls	30.85 **	1.02	29.46 **	1.27
	Total	28,54	0.66	25.60 *,**	0.81
Total test Score	boys	73.93	1.92	72.36	1.95
	Girls	82.85 **	1.40	77.21 *	1.79
	Total	78.17	1.27	74.14	1.41

Note: SEM: Standard Error of Mean; * different to semester 1 (*p* < 0.05); ** different to boys (*p* < 0.05).

## Data Availability

The data presented in this study are not available in accordance with Regulation (EU) of the European Parliament and of the Council 2016/679 of 27 April 2016 regarding the protection of natural persons with regard to the processing of personal data and the free circulation of these data (RGPD).
